# Principles of Molecular Pathology

**DOI:** 10.1038/sj.bjc.6602482

**Published:** 2005-03-22

**Authors:** K Stoeber

**Affiliations:** 1 University College London, UK

Molecular pathology, a rapidly expanding discipline connecting pathology and molecular biology, is providing a deeper insight and understanding of the molecular basis of the aetiology and pathogenesis of human disease. This well-laid-out book covers the basic principles of molecular pathology, explains the most important molecular diagnostic techniques in user-friendly language, and describes their applications across a broad range of human diseases and problems, including cancer, hereditary disorders, identity testing, and infectious diseases. The book is divided into 11 comprehensive yet concise chapters, each providing extensive bibliographic references to the primary literature for those wanting to delve deeper into the subject.

In the first four chapters the reader is introduced to basic concepts of gene structure and function, epigenetics, Mendellian and non-Mendellian patterns of inheritance, population genetics, types of mutations and chromosomal abnormalities, and modern molecular diagnostic techniques. In chapter five, common inherited diseases such as cystic fibrosis and Huntington's disease are discussed as examples of molecular screening for specific mutated genes or linked DNA polymorphisms now commonplace in clinical laboratories. The material covered in this chapter also includes testing for common mitochondrial and metabolic disorders, and the implementation of newborn and carrier screening programmes. In chapters six, seven and eight, basic concepts of tumour biology are covered, including oncogenes and tumour suppressor genes, programmed cell death (apoptosis) and telomere maintenance, and are reviewed in the context of molecular detection of gene rearrangements, tissue-specific gene transcription and oncogene activation in selected sporadic cancers, haematological malignancies, and familial cancer syndromes. Chapter nine provides a useful introduction to the field of pharmacogenetics and includes examples of genes demonstrating pharmacokinetic and pharmacodynamic variation together with their phenotyping. Chapter ten describes the differentiation of individuals from one another by ‘DNA fingerprinting’ for purposes of paternity testing, forensic sample identification, and bone marrow engraftment monitoring. The final chapter discusses the diagnosis and monitoring of human immunodeficiency virus and hepatitis C virus infections as examples of how DNA probes for viruses, bacteria, and parasites promise to revolutionise the practice of medical microbiology.

Overall the book provides an excellent overview of the ongoing molecular revolution that is now transforming pathology and the practice of laboratory medicine. As well as providing a basic reference text for staff in clinical molecular diagnostic laboratories, it also forms a good basis for tutorials and exam preparations. I would highly recommend this book to scientists and health-care professionals working in the field of pathology, including pathology residents, clinicians, and medical students.

